# Evaluating the Influence of Trap Type and Crop Phenological Stage on Insect Population Diversity in Mediterranean Open-Field Tomatoes

**DOI:** 10.3390/insects17010036

**Published:** 2025-12-26

**Authors:** Nada Abdennour, Mehdia Fraj, Ramzi Mansour, Amal Ghazouani, Ahmed Mahmoud Ismail, Hossam S. El-Beltagi, Mohamed M. El-Mogy, Sherif Mohamed El-Ganainy, Wael Elmenofy, Mohamed J. Hajjar, Shimat V. Joseph, Sabrine Attia

**Affiliations:** 1 Laboratory of Bioaggressors and Integrated Pest Management in Agriculture (LR14AGR02), National Agronomic Institute of Tunisia (INAT), University of Carthage, 43 Avenue Charles Nicolle, Cité Mahrajène, Tunis 1082, Tunisia; abdennournada11@gmail.com (N.A.); amal.ghazouani@inat.ucar.tn (A.G.); 2 Laboratory of Diversity, Management and Conservation of Biological Systems (LR18ES06), Faculty of Sciences of Tunis, University of Tunis El Manar, Tunis 2092, Tunisia; frajmehdia@gmail.com; 3 Section of Biological Sciences, ISEP—BG Soukra, University of Carthage, 49, Avenue 13 Août, La Soukra 2036, Tunisia; ramzi_mansour82@yahoo.co.uk; 4 Pests and Plant Diseases Unit, College of Agricultural and Food Sciences, King Faisal University, Al-Ahsa 31982, Saudi Arabia; 5 Agricultural Biotechnology Department, College of Agricultural and Food Sciences, King Faisal University, Al-Ahsa 31982, Saudi Arabia; helbeltagi@kfu.edu.sa; 6 Department of Arid Land Agriculture, College of Agricultural and Food Sciences, King Faisal University, Al-Ahsa 31982, Saudi Arabia; elmogy@kfu.edu.sa (M.M.E.-M.); salganainy@kfu.edu.sa (S.M.E.-G.); welmenofy@kfu.edu.sa (W.E.); mhajjar@kfu.edu.sa (M.J.H.); 7 Department of Entomology, University of Georgia, 1109 Experiment Street, Griffin, GA 30223, USA; svjoseph@uga.edu

**Keywords:** PAN trap, malaise trap, insect diversity, bioindicators, monitoring tool, tomato integrated pest management

## Abstract

The relationship between insect diversity and crop production has been of continuous scientific interest. Understanding insect community dynamics using various sampling and monitoring methods at different crop phenology stages is crucial for enhancing pest management and ecosystem service functioning. The present study assessed the influence of four trap types (Blue, Yellow, White, and Malaise) applied at four tomato developmental stages (start of planting, flowering, flowering fruit development and harvest) on insect diversity in northeastern Tunisian open-field conditions. A total of 1771 insect individuals belonging to seven orders and 31 families were trapped, with the order Hymenoptera being the most common in the sampled plots, which was represented by 25 families. Trap type exerted a strong effect on both abundance and alpha diversity parameters. Yellow pan traps showed the highest diversity, with family richness (S) ranging from 1 to 16, Shannon diversity (H) reaching 2.54, Simpson (Is) diversity ranging from 0.72 to 0.90 and Pielou’s evenness (J) ranging from 0.83 to 0.98. Blue and white traps displayed intermediate diversity (Blue: S = 6 and H = 1.7; White: S = 7 and H = 1.6), while Malaise traps captured the least diverse assemblages (S = 4, H = 1.2 and Is = 0.65). These differences were highly significant (*p* < 0.05). Phenological stage significantly structured Hymenoptera diversity. Richness peaked at the start of planting (S = 1–16 and H up to 2.54) and declined sharply at harvest (S = 1–6). Pollinator families (Apidae, Halictidae, Megachilidae) were the most abundant during flowering, whereas parasitoid families (Braconidae, Eulophidae) dominated during the fruit development stage. Beta diversity analyses (NMDS, stress = 0.25) and PERMANOVA showed that trap type and phenological stage jointly explained 15.5% of the variation in community composition (R^2^ = 0.155, *p* = 0.014). Although a strong taxonomic overlap among traps was observed, Indicator Value analysis revealed significant trap-specific associations, including the family Andrenidae with Blue traps and the family Scoliidae with White and Yellow traps. Overall, the results of the present study demonstrate that both trap type and crop phenology significantly influence insect population diversity. A multi-trap sampling strategy combining colored pan traps and Malaise traps could be recommended to accurately characterize insect communities and associated ecosystem services in Mediterranean open-field tomato systems.

## Simple Summary

We evaluated how different trap types and phenological stages of open-field tomato crops affect the diversity of insects belonging to the order Hymenoptera in southern Mediterranean conditions. We found that colored pan traps, especially the yellow ones, captured the highest diversity of insects, while Malaise traps captured fewer but different insect groups. Insect diversity was highest at the beginning of the crop cycle and during flowering and declined sharply at harvest. Pollinating insects belonging to the families Apidae, Halictidae, and Megachilidae were the most abundant during flowering, whereas parasitoid wasps belonging to the families Braconidae and Eulophidae were more commonly found during the fruit development stage. These results show that using a combination of trap types and considering the timing of crop development stage are essential to accurately monitor hymenopteran beneficial insect communities and to improve sustainable management of tomato agroecosystems.

## 1. Introduction

Agricultural intensification has profoundly reshaped insect community structure in crop systems, disrupting the ecological balance that sustains key ecosystem services such as pollination and environmentally sound pest control. The conversion of diversified agroecosystems into simplified monocultures, coupled with heavy reliance on synthetic pesticides and fertilizers, has reduced habitat heterogeneity and resource availability, thereby threatening beneficial arthropod diversity [1,2]. This simplification not only alters food web complexity but also weakens ecosystem resilience to pest outbreaks and environmental change. Understanding the factors that govern the maintenance or restoration of beneficial insect assemblages is therefore critical for designing sustainable agricultural systems, particularly in Mediterranean regions where climatic variability and continuous cropping intensify ecological pressures [3,4].

A major challenge in studying insect biodiversity in agroecosystems lies in the strong influence of sampling design on community characterization. Different trap types exhibit distinct selectivity toward functional guilds due to variations in behavioral and morphological traits of insects. Malaise traps effectively intercept flying and dispersing taxa such as parasitoids and predators, whereas colored pan traps mainly attract visually oriented flower-visiting taxa, notably pollinators and some dipteran insects [5,6,7]. Because each method captures a partial representation of the community, single-trap approaches may underestimate true taxonomic and functional diversity [8]. Furthermore, trap efficiency interacts with crop phenology, as changes in floral resources, canopy structure, and pest density throughout the growing cycle affect the detectability and abundance of insect guilds [9,10]. However, few studies have simultaneously evaluated the combined effects of trap type and phenological stage in the same crop system, particularly under Mediterranean field conditions.

Crop phenology itself exerts a strong structuring effect on insect communities by determining temporal fluctuations in resource availability and pest-host interactions [11,12]. In tropical and Mediterranean systems where production often occurs year-round, temporal constraints on insect populations are relaxed, potentially favoring generalist taxa over specialists [13]. This dynamic may lead to altered predator–prey and pollinator–plant relationships compared to temperate systems, where seasonal turnover imposes stronger selective filters. Consequently, the ecological and methodological frameworks developed for temperate agroecosystems may not accurately predict biodiversity patterns or biocontrol potential against key pests in Mediterranean tomato production systems [14,15,16].

Tomato (*Solanum lycopersicum* L.) is one of the most widely cultivated vegetable crops worldwide and supports diverse insect assemblages, including insect pests (Gelechiidae, Aleyrodidae), predators (Coccinellidae, Syrphidae, Chrysopidae), parasitoids (Braconidae, Eulophidae), and pollinators (Apidae, Halictidae). These communities fluctuate across developmental stages due to changing habitat structure and pest abundance, notably for both insect pests *Tuta absoluta* (Meyrick) (Lepidoptera: Gelechiidae) and *Bemisia tabaci* (Gennadius) (Hemiptera: Aleyrodidae) [17,18,19,20,21,22]. In Tunisia, like other southern Mediterranean countries, tomato is cultivated across a wide range of agro-climatic zones, offering an ideal model for examining how trap selectivity and crop development stages (phenological cycle) jointly influence insect community dynamics under open-field Mediterranean conditions [14,23,24]. These species were chosen as bioindicators because of their ecological significance, their responsiveness to environmental changes, and their ability to reflect the overall health and dynamics of the agroecosystem.

In this context, the present research aims to (i) evaluate how different sampling methods (Malaise traps vs. pan traps) affect the characterization of insect communities in tomato fields in northeastern Tunisia, (ii) examine the influence of tomato phenological stages on insect diversity, community composition, and functional guild structure, and (iii) identify bioindicator taxa suitable for monitoring beneficial insects in Mediterranean tomato production systems. This study places particular emphasis on the order Hymenoptera, a taxonomically and functionally pivotal group that includes both key parasitoids essential for biological control and vital pollinators contributing to sustainable crop productivity. By integrating methodological and ecological practical dimensions, this work aims to advance robust, ecologically grounded monitoring frameworks that support biodiversity-based pest management in Mediterranean agro-ecosystems.

## 2. Materials and Methods

### 2.1. Study Sites

The study was conducted in 2024 in three tomato fields in northeastern Tunisia. These fields are located in the National Agronomic Institute of Tunisia in the region of Great Tunis (N 36.83122°, E 10.183615°; an agroecological ecosystem cultivated with “tomato + alfalfa”), in Henchir Kort in the Cap-Bon region (N 36.516330°, E 10.637768°; an agroecological ecosystem cultivated with “tomato + alfalfa”) and in Khelidia in the region of Great Tunis (N 36.655438°, E 10.215350°; a conventional field cultivated with “tomato”). The type of climate is Mediterranean, characterized by warm and dry summers and cool and wet winters.

### 2.2. Sampling Procedures

Sampling was conducted using two complementary passive collection methods: Malaise traps and PAN traps. The first type of traps was employed to capture flying insects such as those belonging to the order Hymenoptera, while the second types were used to sample, for example, bees (Apoidea), parasitoids and predatory insects. The Malaise traps consisted of tent-like structures designed to intercept flying insects, which were then funneled into a collecting bottle containing a preservative. PAN traps consisted of colored bowls (Yellow, White and Blue) painted with UV-bright colors to attract insects with different color preferences. Each bowl was filled with water and a few drops of detergent to reduce surface tension. Both trapping methods were operated simultaneously for standardized sampling periods of seven days and repeated weekly throughout the tomato crop cycle (start of planting, flowering, flowering fruit development and harvest stages). Individuals were preserved in ethanol, counted and their families were identified using a Leica MS5 Stereo microscope (Leica Microsystems GmbH, Wetzlar, Germany).

### 2.3. Data Analysis

Data analysis was performed to assess insect community composition, total abundance (N) and mean relative abundance across trap types and tomato crop phenology (development) stages. Diversity patterns were evaluated using several alpha diversity indices, including species richness (S), Shannon–Weaver (H), Simpson (Is) and Pielou evenness (J), to assess evenness and sampling completeness. All data were analyzed for normality and homogeneity by the Shapiro and Levene test, respectively. Statistical differences in individual distributions among insect orders and families were tested using Chi-square tests. For comparisons of insect relative abundances and diversity indices among trap types and crop development stages, non-parametric Kruskal–Wallis tests were applied, followed by Dunn’s post hoc tests for pairwise comparisons when significant differences were detected. Non-metric Multidimensional Scaling (NMDS) ordinations were generated to visualize differences in community composition, while Venn diagrams illustrated the overlap of insect families among traps and crop development stages. To explore patterns in community composition matrices and to evaluate the relative effects of trap type and tomato growth cycle on the insect families, PERMANOVA (Permutational Multivariate Analysis of Variance) was performed based on Bray–Curtis and Jaccard distance. ANOSIM (Analysis of Similarities) was also used to assess group separation. The Indicator Value procedure (IndVal) was used to characterize insect families for each type of traps or crop development stage. The characteristic families received the highest value (100) when all individuals of a family were found in a single trap type or crop cycle of development (high specificity) and when the species occurred at all farms of this type (high fidelity) [25]. All figures and statistical analyses were performed using R v4.5.0 Software [26] and significance levels were set at *p* < 0.05.

## 3. Results

### 3.1. Global Insect Community Composition

A total of 1771 insects were collected during the survey, spanning 7 orders and 31 families. Among these, the order Hymenoptera had the highest count of families (25 families) and accounted for the largest share of insect individuals (1467 individuals). In contrast, the orders Neuroptera and Odonata exhibited lower counts of families (1 family each) and insect individuals (1 and 2 individuals, respectively) (Table 1). The distribution of individuals across the insect orders was statistically different from an expected even distribution (χ^2^ = 6912.4; df = 6; *p* < 2.2 × 10^−16^).

Given the dominance of insects belonging to the order Hymenoptera in terms of both abundance and family richness, and considering their importance as major natural enemies and pollinators, we conducted a more detailed analysis of this order.

### 3.2. Hymenoptera Community Composition

**Figure 1:**
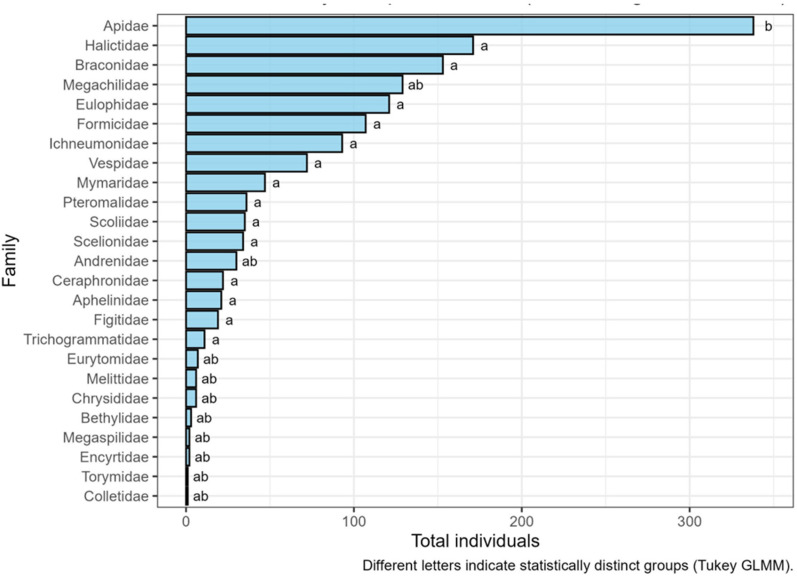
Distribution of the total number of individuals per Hymenoptera family. Different letters indicate significant differences based on a Generalized Linear Mixed Model (GLMM) followed by a Tukey post hoc test (*p* < 0.05).

The distribution of individuals across Hymenoptera families was calculated to examine differences in total abundance. The barplot of total number of individuals and the GLMM (Figure 1) showed that the family Apidae had the highest abundance (338 individuals, group b), while other families such as Braconidae, Formicidae, Halictidae, Ichneumonidae, Mymaridae, Pteromalidae, Scelionidae, Scoliidae, Trichogrammatidae and Vespidae were also abundant but not significantly different from each other (group a). Some families, including Andrenidae, Bethylidae, Chrysididae, Megachilidae and Melittidae, shared intermediate significance (group ab), indicating that they were not significantly different from either the a or b groups (*p* > 0.05).

### 3.3. Mean Number of Individuals Linked to Traps Types and Tomato Development Stages

Differences in the total and mean abundance of Hymenoptera families varied among trap types and crop development stages.

**Figure 2:**
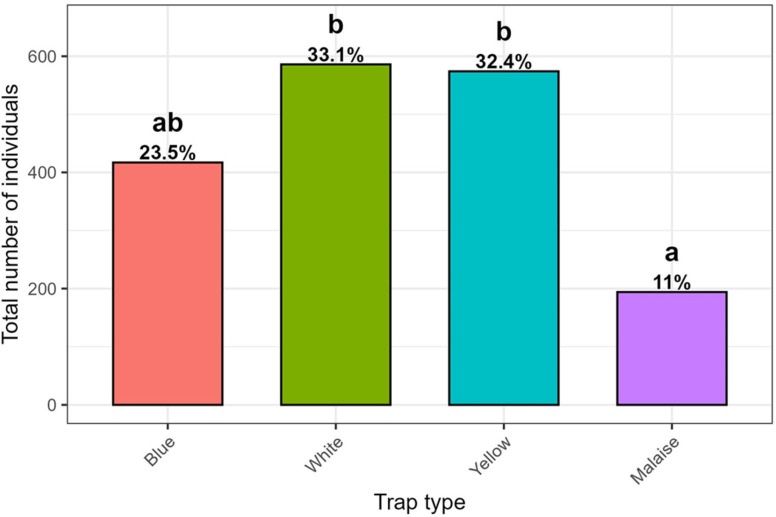
Total number (and %) of insect individuals in the different trap types. Different letters indicate significant differences based on a Kruskal–Wallis (KW) followed by a Dunn’s test, *p* < 0.05.

Regarding trap types, the highest numbers of insect individuals were captured in the White and Yellow traps (N = 586 (33.1%) and N = 574 (32.4%), respectively), followed by Blue traps (N = 417, 23.5%), whereas the lowest number of specimens was recorded in Malaise traps (N = 194 (11%)) (Figure 2). The Kruskal–Wallis followed by the Dunn’s post hoc tests revealed highly significant differences in total number of individuals among Yellow, White and Malaise traps (*p* < 0.05).

Trap type also influenced the composition of captured Hymenoptera families (Table 2). Several families were captured across all trap types, including Aphelinidae, Apidae, Braconidae, Ceraphronidae, Eulophidae, Formicidae, Halictidae, Ichneumonidae, Mymaridae, Pteromalidae, Scelionidae, and Trichogrammatidae. In contrast, some families were specific to certain traps: for example, Andrenidae and Megachilidae were mainly captured in Blue traps, while Colletidae and Megaspilidae were mainly found in Yellow traps.

Differences in the composition of Hymeoptera families among traps reflected variations in functional group abundances. Blue traps showed higher abundances of Andrenidae (4.50 ± 2.81) and Megachilidae (4.00 ± 3.71), highlighting their attractiveness to solitary bees, while parasitoids such as Trichogrammatidae were present in low numbers (1.00 ± 0.00). White traps predominantly captured pollinators, with Apidae showing the highest mean relative abundance (6.12 ± 6.42), whereas other families like Melittidae were less abundant (1.00 ± NA). Yellow traps attracted a mix of pollinators and parasitoids, particularly Colletidae (4.76 ± 3.17) and Eulophidae (3.33 ± 2.74). In contrast, Malaise traps captured fewer bees, but Formicidae were more abundant (3.80 ± 5.47), along with small parasitoids such as Mymaridae (2.20 ± 1.32). The Kruskal–Wallis tests confirmed that several of the differences in insect family abundances among trap types were statistically significant. In particular, Colletidae (χ^2^ = 19.08, df = 3, *p* = 0.00026), Apidae (χ^2^ = 15.22, df = 3, *p* = 0.00164), Scelionidae (χ^2^ = 8.03, df = 3, *p* = 0.045), and Mymaridae (χ^2^ = 8.19, df = 3, *p* = 0.042) showed significant variation between trap types. Post hoc Dunn tests revealed that for Apidae, abundances in White traps were significantly higher than in Blue traps (*p* = 0.0017) and Malaise traps (*p* = 0.019), while for Colletidae, Yellow traps captured significantly more individuals than Blue traps (*p* = 0.00017) and White traps (*p* = 0.0176). These results indicate that trap color strongly influences the capture of key pollinator and parasitoid families, with White traps favoring Apidae and Yellow traps favoring Colletidae.

**Figure 3:**
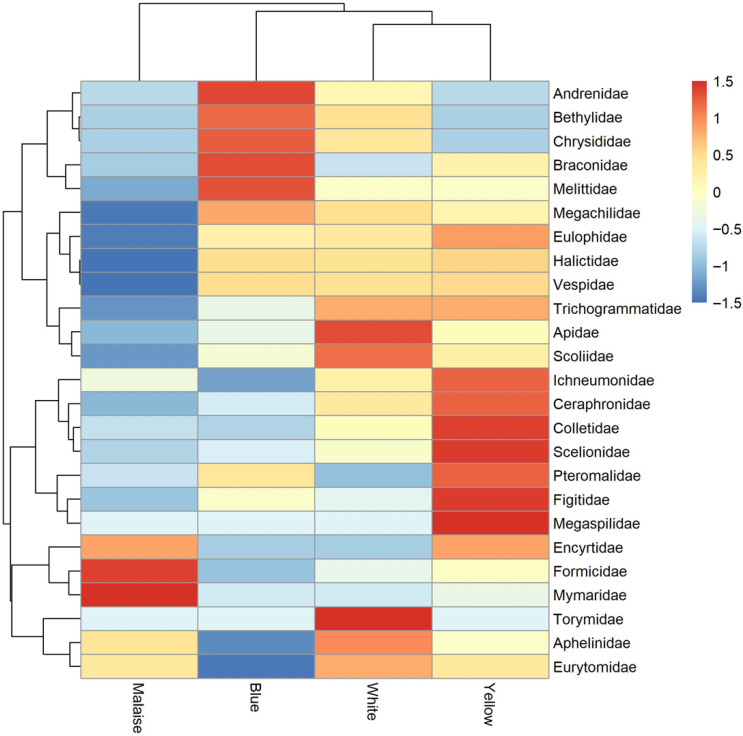
Heatmaps of insect family abundances across trap types (maximum of abundance is colored with red, while minimum of abundance is colored with blue).

Hierarchical clustering of Heatmap analysis (Figure 3; vertical dendrogram) showed two distinct functional groups based on their differential responses to trap types. The first cluster predominantly comprised pollinating bees (Andrenidae, Melittidae, Megachilidae and Halictidae) along with small predatory and parasitoid wasps (Bethylidae, Chrysididae, Braconidae, Eulophidae and Vespidae). These families exhibited strong attraction to Malaise and Blue traps, suggesting a common behavioral response to metallic visual cues typically associated with floral resources. The second cluster consisted primarily of specialized parasitoids of varying sizes, including micro-hymenopteran egg parasitoid (Trichogrammatidae and Scelionidae), larger parasitoids (Ichneumonidae and Scoliidae), and other families (Pteromalidae, Figitidae, Encyrtidae and Mymaridae). The horizontal dendrogram demonstrated a clear separation between two trap categories exhibiting contrasting capture efficiencies. The Malaise traps formed an isolated cluster, diverging significantly from the three other trapping methods (Blue, White and Yellow), which were grouped together in a second cluster. The Malaise traps predominantly captured Formicidae and Encyrtidae (dark red indicating high abundances). This dichotomy suggests that the Malaise trap operates through a fundamentally different attraction mechanism, preferentially capturing families from the first vertical cluster (pollinators and small predators) while showing low efficiency for specialized parasitoids (blue color in Heatmap). Conversely, within the second trap cluster, the Yellow trap was notably effective in capturing parasitoid families (Scelionidae, Pteromalidae, Figitidae and Cerphronidae), whereas Blue (Andrenidae, Braconidae) and White (Apidae, Torymidae) traps exhibited intermediate profiles. These findings demonstrate the absence of a universal trapping method and underscore the necessity of employing a multi-trap approach combining minimally a Malaise-type and a Yellow-type trap to achieve representative sampling of hymenopteran diversity, avoiding substantial taxonomic bias in biodiversity surveys.

**Figure 4:**
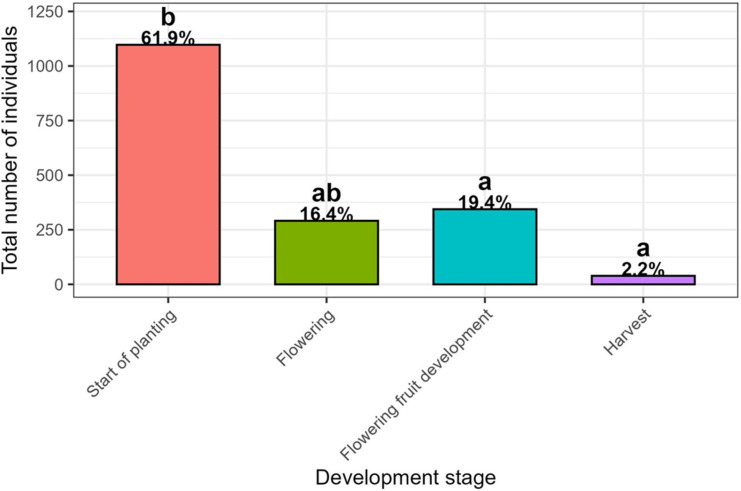
Total number (and %) of insect individuals linked to tomato development stages. Different letters indicate significant differences based on a Kruskal–Wallis (KW) followed by a Dunn’s test, *p* < 0.05.

On the other hand, the total abundance of the family Hymenoptera varied significantly among tomato development stages (Figure 4). The highest number of insect individuals was recorded at the start of planting (N = 1097; 61.9%), followed by the flowering fruit development and flowering stages (N = 344; 19.4% and N = 291; 16.4%, respectively). The lower individual counts were recorded at the harvest stage (N = 39; 2.2%). The Kruskal–Wallis followed by the Dunn’s post hoc tests showed highly significant differences in total individuals counts among start of planting, flowering fruit development and harvest stages (*p* < 0.05). Variations in the composition of Hymenoptera families were studied across tomato growth stages (Table 3), reflecting shifts in the mean abundance of functional groups. At the start of planting stage, parasitoids such as Braconidae (3.02 ± 2.29) and pollinators like Colletidae (3.41 ± 3.30) were predominant, while Megachilidae (2.59 ± 1.77) and Halictidae (3.57 ± 3.39) showed moderate representation. During the flowering stage, the abundance of Megachilidae increased remarkably (6.11 ± 4.70), together with Colletidae (3.08 ± 2.39) and Apidae (2.85 ± 3.62), indicating enhanced pollinator activity. The flowering fruit development stage was characterized by high occurrences of Eulophidae (3.71 ± 3.17) and Braconidae (1.33 ± 0.65), reflecting a rise in parasitoid diversity, while pollinators such as Halictidae (2.06 ± 1.09) remained stable. At the harvest stage, overall insect abundance declined, with only Vespidae (2.75 ± 2.06) and Scelionidae (2.00 ± NA) maintaining moderate presence. These variations suggest that pollinator activity peaked during flowering, while parasitoid families were more represented during the fruit development stage, before declining at harvest. Significant differences (Kruskal–Wallis completed by Dunn test) were observed among insect families across crop development stages. Notably, Braconidae showed significant differences between start of planting and flowering fruit development (*p*_adj = 0.0012), Megachilidae between flowering and flowering fruit development (*p*_adj = 0.048), Scoliidae between start of planting and flowering (*p*_adj = 0.032), and Syrphidae between start of planting and flowering fruit development (*p*_adj = 0.016).

**Figure 5:**
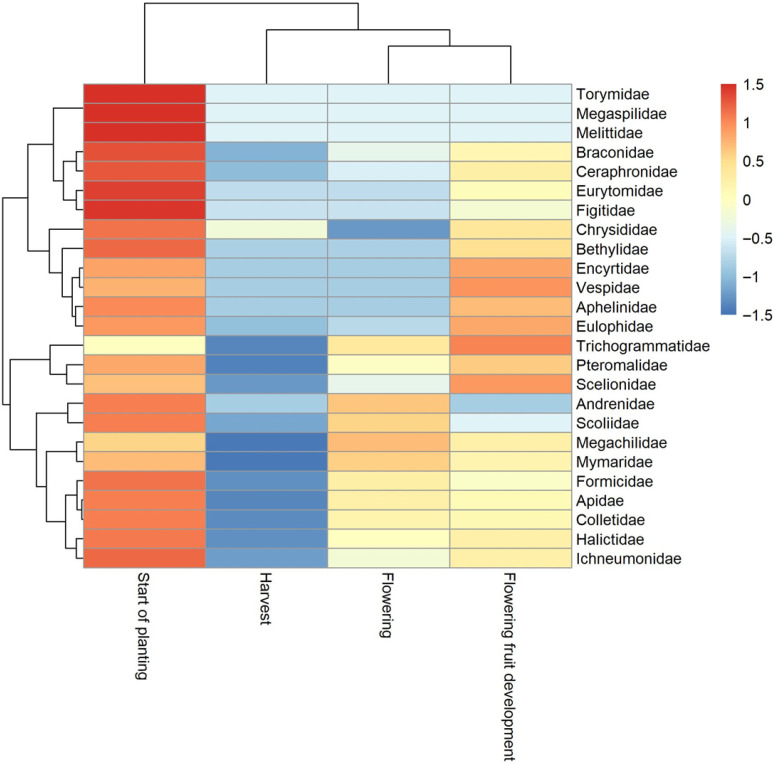
Heatmaps of Hymenoptera family abundances across tomato development stages (maximum and minimum of abundance colored with red and blue, respectively).

Heatmap analysis (Figure 5) of trapped family distributions across crop development stages revealed distinct temporal patterns in community composition.

The vertical dendrogram identified two major functional groups exhibiting contrasting phenological patterns. The first cluster, comprising predominantly parasitoid wasps and predators (Torymidae, Megaspilidae, Braconidae, Ceraphronidae, Eurytomidae, Figitidae, Chrysididae, Bethylidae, Encyrtidae and Eulophidae), showed maximal abundance during the start of planting stage (high positive values) with subsequent decline through harvest and flowering periods (negative values). This early-season emergence likely reflects synchronization with early pest populations and spring-active hosts, suggesting a critical role in biological control during crop establishment. In contrast, the second cluster displayed a more complex temporal pattern, characterized by moderate activity at start of planting, pronounced depression during harvest and recovery or peak activity during reproductive stages. This group included pollinating bees (Apidae, Halictidae, Megachilidae, Andrenidae and Colletidae) showing maximal abundance during flowering and flowering fruit development stages, alongside parasitoid families (Trichogrammatidae, Pteromalidae and Scoliidae) exhibiting bimodal activity peaks at start of planting and flowering fruit development stages, with pronounced depression during harvest. This finding suggests multivoltine life cycles or dual host exploitation strategies aligned with both early-season and late-season pest populations.

The horizontal dendrogram emphasized this phenological structuring, with start of planting forming an isolated cluster distinctly separated from the grouped harvest, flowering and flowering fruit development stages. This bifurcation underscores a fundamental shift in community composition from early-season parasitoid dominance to a more diverse assemblage of pollinators and late-season natural enemies. These findings emphasize the necessity of implementing phenologically targeted management strategies that preserve natural enemy populations during both crop establishment and fruit development while protecting pollinator activity during flowering to optimize complementary pest control and pollination services throughout the entire growing season.

### 3.4. Alpha Diversity Analyses

**Figure 6:**
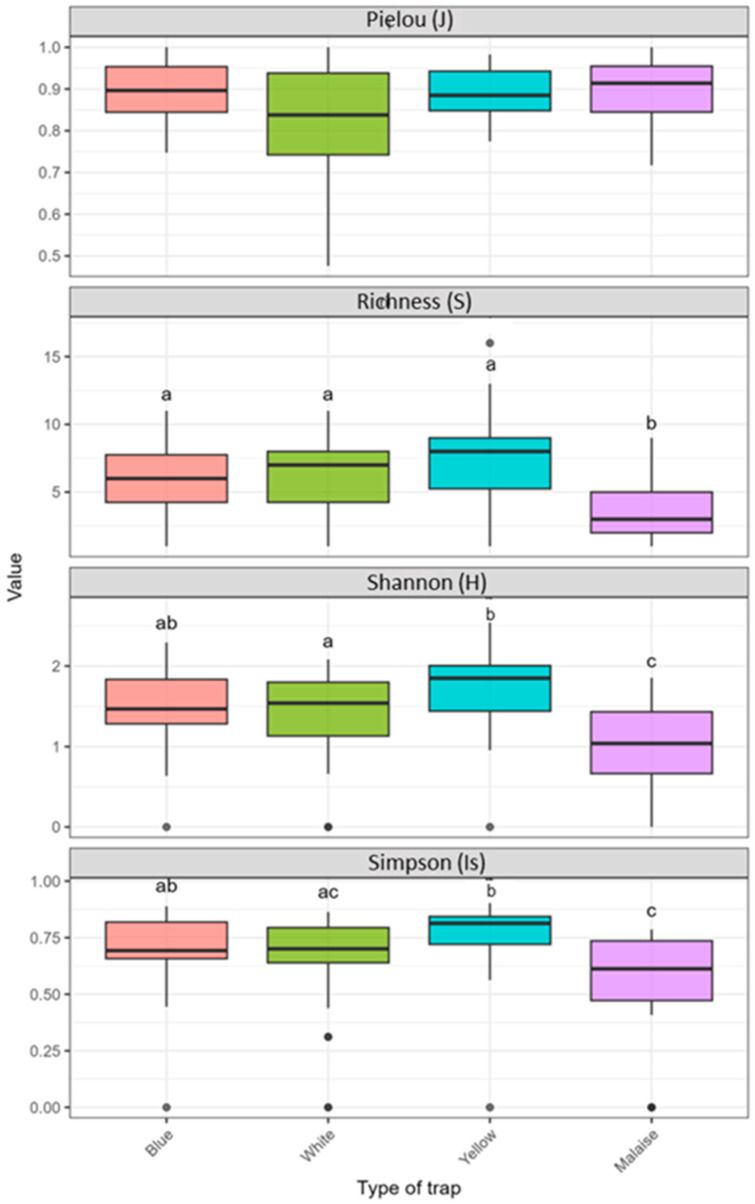
Alpha diversity indices of Hymenoptera communities according to trap types. Box plots show median, quartiles and variability for each diversity index. Kruskal–Wallis (KW) *p*-values were shown above each trap; different letters indicate significant differences (Dunn’s test, *p* < 0.05).

Alpha diversity exhibited variations related to traps and tomato development stages. According to traps types, significant differences in community structure was observed (Figure 6). In particular, Yellow traps yielded the greatest species richness as well as the highest diversity indices, thereby highlighting their superior efficiency in capturing diverse assemblages. Richness ranged from 1 to 16 with a high median of 8 families, while Shannon index value varied between 0 and 2.54 with a median close to 1.9. Furthermore, Simpson values were generally high (from 0.72 to 0.90; median = 0.84) and Pielou’s evenness index was also high (varied between 0.83 and 0.98; median = 0.92), jointly indicating both high diversity and relatively even community structure.

Blue traps exhibited intermediate diversity patterns. S ranged between 1 and 11, with a median of approximately 6 families, while H values varied from 0 to 2.29 (median ≈ 1.6–1.8). In addition, Is indices extended from 0.44 to 0.89 with median equal to 0.80 and J evenness remained high but slightly more variable (0.74–1.00; median = 0.90). Similarly, White traps showed intermediate levels of diversity, with S ranging from 1 to 11 and a median around 7 families. Correspondingly, H values fluctuated between 0 and 2.08 (median = 1.6), Is ranged from 0.31 to 0.86 (median = 0.76) and J indices varied between 0.48 and 1.00 (median = 0.9).

Controversially, the lowest diversity levels were recorded in Malaise traps. S did not exceed 9, with a lower median (4 families). Likewise, H ranged from 0 to 1.85 (median = 1.2), while Is indices extended from 0 to 0.78 (median = 0.65). Additionally, J evenness exhibited marked heterogeneity and values ranged from 0.72 to 1.00 (median = 0.85). These results demonstrate that Yellow traps consistently outperformed the other trap types, capturing not only greater family richness but also more balanced assemblages.

Statistical analysis (Kruskal–Wallis test) revealed highly significant differences in S (χ^2^ = 20.55, df = 3, *p* = 0.00013), H (χ^2^ = 21.47, df = 3, *p* = 0.00008), Is (χ^2^ = 23.40, df = 3, *p* = 0.00008) among trap types, mainly driven by contrasts between Malaise and Yellow traps (post hoc Dunn’s test, *p* < 0.05).

**Figure 7:**
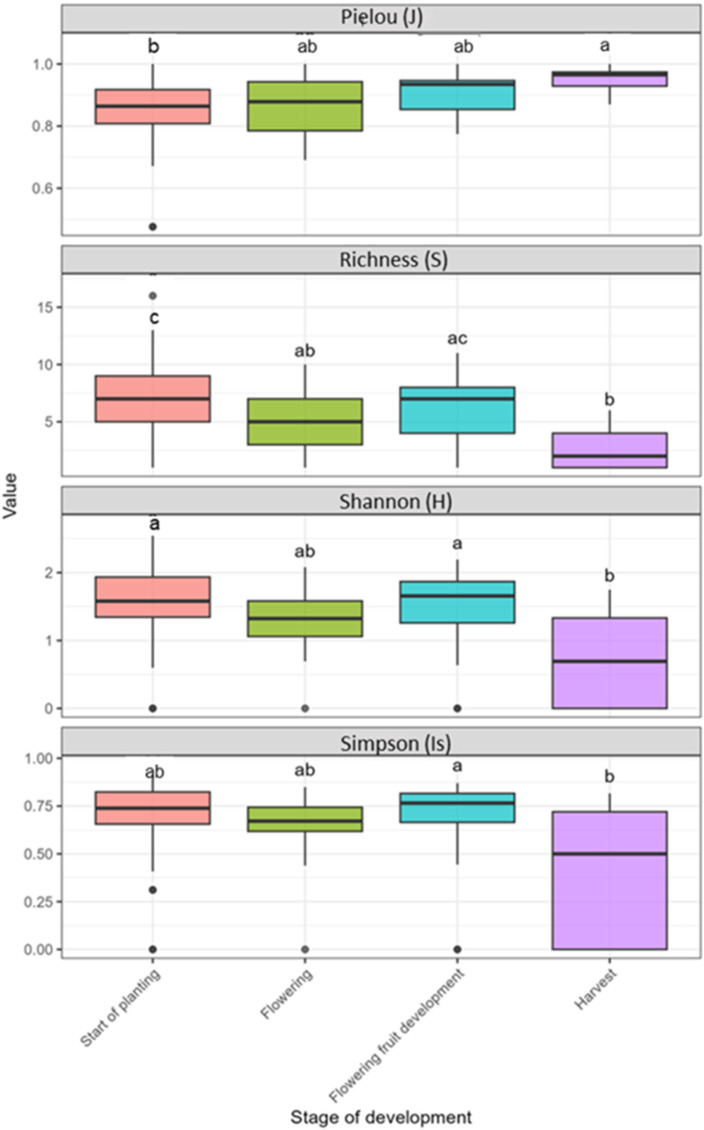
Alpha diversity indices of insect communities according to crop development stages. Box plots show median, quartiles and variability for each diversity index. Kruskal–Wallis (KW) *p*-values were shown above each cycle; different letters indicate significant differences (Dunn’s test, *p* < 0.05).

Temporal fluctuation in alpha diversity was also observed across all tomato stages of development (Figure 7). Overall, species richness (S) varied considerably between 1 and 16, reflecting substantial shifts in community structure throughout the crop cycle. At the first stage, S ranged from 1 to 16 (median = 7), with one particularly notable outlier reaching 16 families. Correspondingly, the H index exhibited a wide range from 0 to 2.54 (median = 1.65), while Is index values were generally high and varied between 0.31 and 0.90 (median = 0.76). Furthermore, J evenness remained consistently high and reached 1.00 (median = 0.87), indicating structurally complex and relatively evenly distributed communities at the onset of the crop cycle. Subsequently, the flowering stage displayed intermediate diversity levels. S ranged from 1 to 10 (median = 5), while H values varied between 0 and 2.08 (median = 1.35) and Is values ranged from 0.44 to 0.85 (median = 0.7). Evenness (J) remained relatively stable (0.69–1.00; median = 0.86), suggesting maintained community structure despite moderate reductions in richness. Additionally, the flowering fruit development stage exhibited diversity parameters and indices comparable to those observed during flowering. Richness (S) ranged from 2 to 11 (median = 7), H varied from 0.64 to 2.19 (median = 1.65) and Is reached 0.87 (median = 0.76). Moreover, evenness remained consistently high (0.77–0.97; median = 0.91), indicating sustained community balance throughout flowering fruit development. In contrast, the harvest stage exhibited significantly lower diversity compared to all earlier developmental stages. Richness declined substantially (from 1 to 6; median = 2), with several samples containing only a single family, thereby representing a simplification of the Hymenoptera community. Shannon diversity was also remarkably reduced (0–1.75; median ≈ 0.65) and Is index revealed pronounced variability (from 0 to 0.82; median = 0.50) with some samples being completely dominated by one or two families. However, despite this overall decline, J evenness remained relatively high in samples with multiple families (0.87–1.00; median = 0.97), suggesting that although fewer families were present at harvest, the remaining Hymenoptera families were still relatively evenly distributed within their respective communities. Kruskal–Wallis followed test indicated significant variation in all Hymenoptera diversity indices linked to crop development stage (*p* < 0.05). Dunn post hoc test revealed that the main differences occurred between start of planting, flowering fruit development and harvest stages.

### 3.5. Community Composition and Beta Diversity Patterns

**Figure 8:**
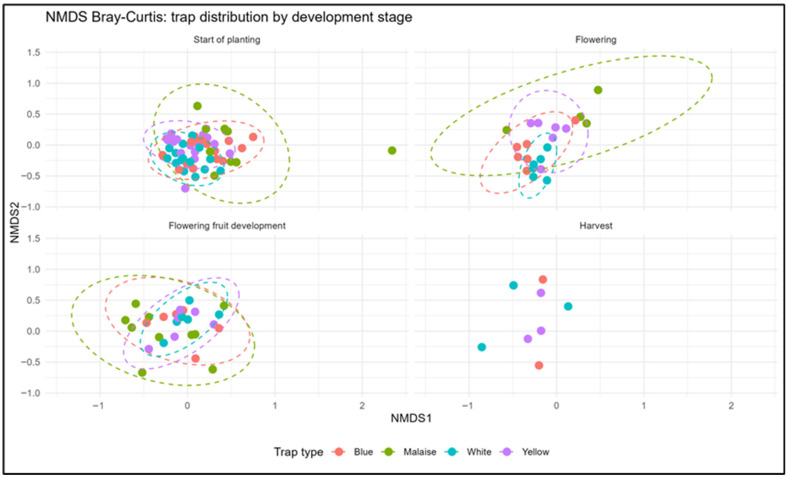
NMDS analysis of insect community similarity among traps and crop development stages based on a Bray–Curtis dissimilarity matrix.

Non-metric multidimensional scaling (NMDS) analysis using Bray–Curtis (Hellinger) and dissimilarity indices (Figure 8) revealed patterns in Hymenoptera community composition across trap types and crop developmental stages (stress = 0.25, k = 2). NMDS axis 1 (NMDS1) captured the main gradient of variation among trap types, while axis 2 (NMDS2) reflected subtler within-stage of development differences. Early-season communities at start of planting stage exhibited high overlap among trap types (NMDS1: −0.27 to 0.78; NMDS2: −0.66 to 0.73) dominated by generalist insect families. As crops progressed through flowering and flowering fruit development stages, trap-specific assemblages began to diverge, particularly Malaise traps, which captured distinct flying Hymenoptera (NMDS1 = 0.46, NMDS2 = −0.95 during flowering). At harvest, trap-type differentiation was maximal: Blue traps (NMDS1 = −0.57 to 0.39; NMDS2 = −0.65 to 0.57), Malaise traps (NMDS1 = −0.64 to 0.42; NMDS2 = −0.44 to 0.71) and White and Yellow traps occupied unique ordination spaces, illustrating functionally specialized late-season communities. Faceted NMDS ordinations by developmental stage highlighted the consistency of trap-specific groupings throughout the cropping cycle. PERMANOVA analysis confirmed that trap type and phenological stage jointly explained 15.5% of the variation in Hymenoptera communities (F_14,98_ = 1.29, *p* = 0.014, R^2^ = 0.155) validating that sampling method consistently structures community composition while temporal dynamics drive gradual differentiation across crop development stages. These results underscore the necessity of using multiple sampling approaches to capture the full spectrum of Hymenoptera diversity in open-field tomatoes.

**Figure 9:**
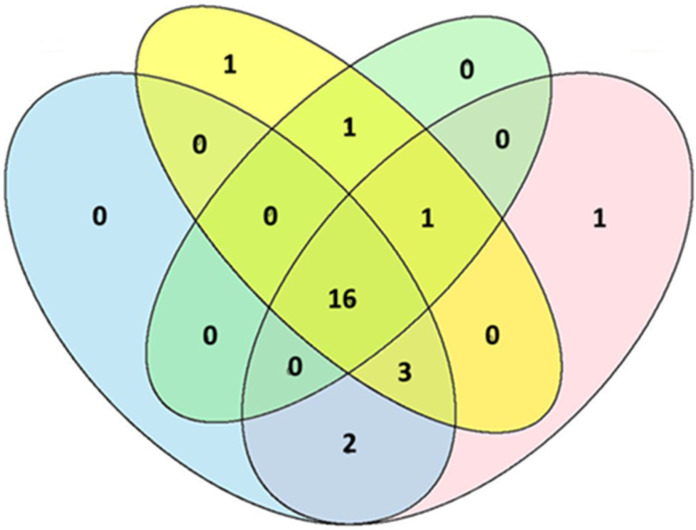
Venn diagrams of Hymenoptera family composition across trap types. Numbers indicate families unique to specific traps and those shared among multiple traps. Blue color: blue traps; yellow color: yellow traps; green color: Malaise traps; pink color: white traps.

Venn diagram analysis (Figure 9) of Hymenoptera composition across the trap types revealed substantial family overlap. Diagram showed that 16 families were consistently captured in all four trap types (Blue, Yellow, Malaise and White), representing a core assemblage of the family Hymenoptera. Most intersections yielded 0–1 unique families, indicating limited trap-specific capture patterns. This high convergence suggests that different trap types sample largely overlapping family’s community, with trap selection driven more by logistical considerations than unique family level taxonomic coverage.

**Figure 10:**
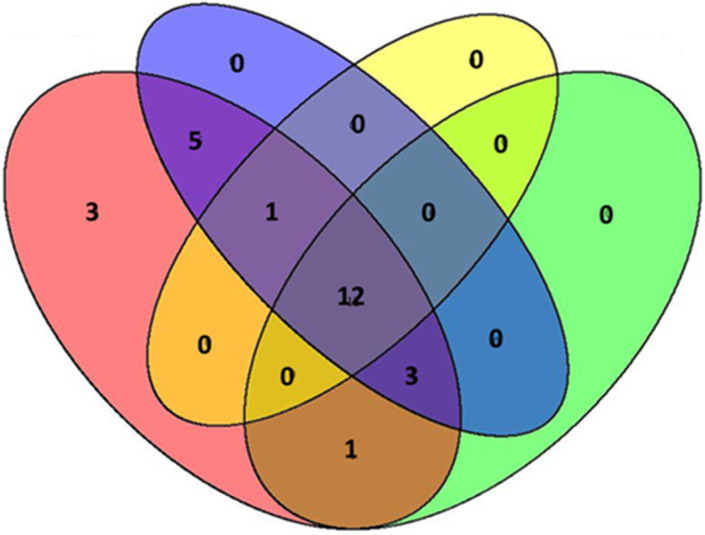
Venn diagrams of Hymenoptera family composition across tomato development stages. Numbers indicate families unique to specific stages and those shared among multiple stages. Pink color: start of planting; blue color: flowering fruit development; yellow color: harvest; green color: flowering.

Temporal variation across crop developmental stages showed moderate stability. Analysis across four crop developmental stages (Figure 10) revealed 12 families consistently present throughout the growing season. The start of planting stage had three unique families, likely representing early-season colonizers. The high number of families present across all stages (*n* = 12) indicates consistent community structure throughout crop development, with only a subset of families showing stage-specific patterns.

### 3.6. Indicator Value (IndVal) Analysis

To verify the patterns observed in the Venn diagram analysis, an Indicator Value (IndVal) analysis was performed (Table 4). The results showed significant and family-specific associations with particular trap groups. Andrenidae was significantly associated with the Blue traps (IndVal = 0.424; *p* = 0.005), while Scoliidae was associated with the White + Yellow trap group (IndVal = 0.482; *p* = 0.039). Several families showed strong indicator values for the Blue + White + Yellow trap group, including Halictidae (IndVal = 0.795; *p* = 0.001), Megachilidae (IndVal = 0.691; *p* = 0.001), Vespidae (IndVal = 0.667; *p* = 0.001) and Eulophidae (IndVal = 0.664; *p* = 0.003). In addition, Ichneumonidae was significantly associated with the Malaise + White + Yellow trap group (IndVal = 0.648; *p* = 0.007), confirming that although overall family composition strongly overlapped among traps, a small subset of families exhibited clear trap-type preferences.

## 4. Discussion

We studied the composition and diversity of insect assemblages related to trap types and tomato development stages. In total, 31 insect families were collected, indicating substantial taxonomic diversity in the tomato agroecosystem. The order Hymenoptera dominated our collections (25 families), a pattern consistent with findings across Mediterranean agroecosystems [27], where parasitoids and predatory wasps constitute the functional backbone of arthropod communities. This dominance reflects the pivotal ecological role of Hymenoptera as primary natural enemies responding to pest pressure in simplified crop systems. However, the balanced representation of secondary families within the Hymenoptera, notably Apidae, Halictidae, and Braconidae indicates functional redundancy in pest suppression mechanisms [28,29], which acts as an ecological buffer against phenological mismatches and environmental perturbations [30].

We found that Malaise traps, despite capturing a lower number of insect individuals and lower diversity indices, were selective for aerial parasitoids and predators. These passive intercept traps are known for their high efficiency in capturing flying insects, especially those that are underrepresented in color-based traps [31,32]. Conversely, pan traps yielded significantly higher abundance and diversity (Shannon and Simpson indices). 

Our results confirmed that trap color strongly influences insect capture due to differences in spectral reflectance and visual attraction [33]. Yellow traps attracted a wide range of generalist pollinators and parasitoids (e.g., Megachilidae, Chrysididae), while White traps were dominated by pollinators such as Apidae. These findings corroborate those of Jaques et al. [34] who demonstrated that trap color significantly affects sample composition, with yellow traps consistently capturing the highest species richness and white traps favoring pollinator-dominated assemblages across diverse agroecosystems [35]. Blue traps, in contrast, predominantly captured communities dominated by pollinators (notably Andrenidae), reflecting their strong efficacy in attracting fast-flying taxa and emphasizing the ecological importance of these pollinators in the agroecosystem. This finding confirms that Blue traps, although more selective, are valuable for detecting specialized functional guilds [36].

Rather than viewing these methods as competing, our hierarchical clustering analysis revealed a clear functional complementarity: Blue and White traps grouped together, while Malaise traps formed a distinct cluster. This pattern suggests that chromatic traps provide overlapping but complementary samples, whereas passive interception traps capture distinct guilds absent in color-based methods [34,36]. The observed functional partitioning by trap type confirms that monitoring design critically shapes ecological interpretations, with major implications for biodiversity assessment and long-term monitoring [37]. Using a single trapping method can lead to systematic underestimation of functional diversity and omission of taxa essential for ecosystem services such as pollination and biological pest control. This study also reinforces previous findings that arthropod community dynamics in tomato fields follow structured temporal patterns linked to crop phenology [38]. Insect community composition shifted predictably with the crop development stages, reflecting temporal specialization of functional guilds. During the initial phase of cultivation, insect abundance and diversity were high, particularly for functionally important families such as Andrenidae, and Apidae, consistent with slower colonization of newly established habitats [39].

As the crop developed, floral resources and prey availability increased, leading to a surge in pollinator (Megachilidae) and Colletidae populations during the flowering stage. This aligns with the notion that nectar and pollen availability enhance pollinator activity [40]. Similarly, high abundances of Eulophidae and Aphelinidae during flowering and fruit development stages indicate that natural enemies especially parasitoids respond to increased host and prey density [41]. The moderate decline of Megachilidae during late fruiting suggests stabilization of pollinator populations after peak flowering. Finally, the sharp reduction in insect abundance at harvest can be attributed to crop senescence and mechanical disturbances, which reduce habitat suitability [42].

In terms of diversity metrics, taxonomic richness and evenness increased from planting then decreased during flowering and fruiting stages, when floral and herbivore resources were most abundant. Diversity then declined sharply at harvest, confirming previous observations linking crop senescence to reductions in resource-dependent taxa [43]. Evenness indices remained relatively stable across phenological stages [44], suggesting that while species composition varied, community structure remained balanced. Heatmap clustering further illustrated these temporal dynamics, insect abundance peaked at the start of planting stage, remained relatively high during flowering and fruit development, and declined remarkably at harvest, reflecting the temporal dynamics of resource availability and habitat suitability throughout the crop cycle [45]. These results emphasize the importance of temporal habitat management in tomato agroecosystems, to sustain beneficial insect populations and their ecosystem services throughout the crop cycle [46]. Capturing functional groups throughout all phenological stages is fundamental because tomato crops exhibit pronounced temporal fluctuations in resource availability and habitat structure, which mechanistically shape the activity, abundance, and assemblage turnover of both pests and beneficial arthropods. Functional guilds do not respond uniformly across the crop cycle: pollinators peak during flowering, herbivores track vegetative growth and fruit formation, and parasitoids closely follow host density patterns. Monitoring these stage-specific trajectories provides a dynamic picture of regulatory processes that cannot be inferred from static sampling approaches. From a pest management perspective, resolving functional-group dynamics allows for the identification of moments when natural biological control is strongest or, conversely, when pest populations are most likely to escape regulation due to phenological mismatches with their natural enemies. Such mismatches are increasingly recognized as key drivers of pest outbreaks in annual cropping systems. By understanding when specific parasitoid or predator guilds are naturally abundant, growers can strategically minimize chemical insecticide applications, reduce mortality of non-target living organisms, and enhance the persistence of beneficial arthropods. Furthermore, incorporating functional-group monitoring into routine surveillance supports a shift toward more predictive, ecologically informed Integrated Pest Management (IPM). It enables managers to anticipate service provision, detect early-warning signals of pest irruptions, and implement interventions that are temporally aligned with the agroecosystem’s intrinsic regulatory capacity. Thus, temporal functional profiling is not only ecologically informative but also operationally indispensable for designing high-precision, sustainable pest control strategies. Multivariate analyses (NMDS) confirmed these trends, revealing significant differences in community composition among trap types and developmental stages. Early in the season, insect communities were relatively homogeneous across traps, but clear separation between Malaise and pan traps emerged during flowering–fruiting, reflecting trap selectivity effects. PERMANOVA results indicated that phenological stage explained 15.5% of the variation in Hymenoptera communities, suggesting that broader ecological drivers (climate, landscape, management) also influence community structure beyond methodological effects [47,48]. Indicator value (IndVal) analysis provided further insight into trap efficiency and functional associations. Ichneumonidae showed the strongest values with the Malaise, White and Yellow traps, confirming the suitability of these trap types for monitoring aerial parasitoids, especially egg parasitoids of lepidopteran pests. This is consistent with the family’s high alpha and beta diversity values and its recognized importance as a bioindicator of parasitoid community dynamics [49,50].

Regarding tomato development stages, Megachilidae exhibited strong association with the flowering stage, confirming their role as key pollinators dependent on floral resource abundance [51]. These solitary bees, known as leafcutter and mason bees, are among the most efficient pollinators, comparable to honey bees for certain crops, due to their specialized pollen-carrying structures and strict dependence on nectar and pollen for larval provisioning. Their peak activity during flowering makes them reliable indicators of reproductive growth phases and floral resource availability. In contrast, Mellitidae showed no association with the flowering stage, further underscoring their value as indicators of floral resource intensity and population sensitivity to temporal resource shifts.

## 5. Conclusions

Overall, insect families belonging to the order Hymenoptera were the most frequently recorded in field sites of the present study. The highest number of insect individuals were captured in White and Yellow traps, compared to Malaise traps. Yellow and Malaise traps mainly attracted predators, while Blue traps mainly attracted pollinators (Andrenide). The highest diversity values of families of the order Hymenoptera were observed in plots where all four traps (Blue, white, Yellow and Malaise) were applied. The present study highlights the ecological significance of integrating multiple trap types and crop phenological stages when monitoring insect biodiversity in tomato agroecosystems in northeastern Tunisia. The combined use of Malaise and color pan traps provides a more comprehensive view of community structure, enabling the detection of both aerial parasitoids and visually oriented pollinators. From a management perspective, our results demonstrate that biodiversity-based monitoring can serve as a powerful diagnostic tool for ecosystem health and functional service delivery. Thus, this research contributes to the development of ecologically informed monitoring frameworks and supports the integration of biodiversity conservation, especially for beneficial hymenopteran insects (pollinators and parasitoids) providing crucial ecosystem services, in Mediterranean tomato production systems.

## Figures and Tables

**Table 1:** Structure of insect communities in all trap types.

Insect Order	Number of Families	Number of Insect Individuals
Hymenoptera	25	1467
Coleoptera	1	200
Hemiptera	1	61
Diptera	1	32
Dermaptera	1	8
Odonata	1	2
Neuroptera	1	1
Total number	31	1771

**Table 2:** Mean abundance of Hymenoptera families related to traps types.

Families	Blue	White	Yellow	Malaise
Andrenidae	4.50 (2.81)	1.00 (0.00)	0	0
Aphelinidae	1.00 (NA)	2.00 (2.24)	1.33 (0.58)	1.50 (1.00)
Apidae	1.56 (1.05)	6.12 (6.42)	2.30 (1.78)	1.89 (1.94)
Bethylidae	1.00 (0.00)	1.00 (NA)	0	0
Braconidae	2.80 (2.42)	2.14 (1.83)	2.29 (1.93)	2.55 (2.25)
Ceraphronidae	1.50 (0.71)	1.50 (0.58)	1.38 (0.52)	1.00 (0.00)
Chrysididae	1.00 (0.00)	1.00 (0.00)	1.00 (NA)	1.00 (NA)
Colletidae	1.33 (1.18)	2.35 (2.40)	4.76 (3.17)	2.44 (1.51)
Encyrtidae	0	0	1.00 (NA)	1.00 (NA)
Eulophidae	2.70 (2.36)	2.21 (1.93)	3.33 (2.74)	1.00 (0.00)
Eurytomidae	0	1.00 (0.00)	2.00 (NA)	1.00 (0.00)
Figitidae	1.00 (0.00)	1.00 (0.00)	2.17 (2.86)	1.00 (NA)
Formicidae	1.64 (1.08)	2.00 (1.47)	1.75 (1.29)	3.80 (5.47)
Halictidae	2.84 (3.44)	2.89 (2.32)	2.52 (2.13)	1.40 (0.55)
Ichneumonidae	1.33 (0.52)	2.56 (2.96)	2.19 (1.40)	2.00 (1.41)
Megachilidae	4.00 (3.71)	2.47 (2.56)	2.00 (1.49)	0
Megaspilidae	0	0	1.00 (0.00)	0
Melittidae	1.33 (0.58)	1.00 (NA)	1.00 (NA)	0
Mymaridae	1.14 (0.38)	1.14 (0.38)	1.12 (0.35)	2.20 (1.32)
Pteromalidae	1.43 (0.53)	1.25 (0.50)	1.88 (0.99)	1.20 (0.45)
Scelionidae	1.00 (0.00)	1.20 (0.45)	2.09 (1.22)	1.00 (0.00)
Scoliidae	1.25 (0.50)	2.10 (1.73)	1.14 (0.38)	1.00 (NA)
Torymidae	0	1.00 (NA)	0	0
Trichogrammatidae	1.00 (0.00)	1.33 (0.58)	1.00 (0.00)	1.00 (NA)
Vespidae	2.00 (1.54)	1.64 (1.01)	1.79 (1.05)	0
Mean abundance	2.12 (2.19)	2.84 (3.68)	2.37 (2.07)	2.04 (2.3)

NA indicates that the standard deviation (sd) could not be calculated because only one individual was recorded for that family per trap type.

**Table 3:** Mean relative abundance (sd) of insect individuals per family linked to tomato development stages.

Families	Start of Planting	Flowering	Flowering Fruit Development	Harvest
Andrenidae	4.00 (2.83)	2.50 (3.00)	0	0
Aphelinidae	1.30 (0.67)	0	2.67 (2.89)	0
Apidae	3.60 (4.93)	2.85 (3.62)	2.40 (1.76)	1.67 (0.58)
Bethylidae	1.00 (0.00)	0	1.00 (NA)	0
Braconidae	3.02 (2.29)	1.00 (0.00)	1.33 (0.65)	1.00 (NA)
Ceraphronidae	1.40 (0.52)	2.00 (NA)	1.25 (0.50)	1.00 (NA)
Chrysididae	1.00 (0.00)	0	1.00 (0.00)	1.00 (NA)
Colletidae	3.41 (3.30)	3.08 (2.39)	2.27 (1.39)	2.00 (0.00)
Encyrtidae	1.00 (NA)	0	1.00 (NA)	0
Eulophidae	2.46 (1.91)	1.50 (1.00)	3.71 (3.17)	1.33 (0.58)
Eurytomidae	1.20 (0.45)	0	1.00 (NA)	0
Figitidae	1.64 (2.11)	0	1.00 (NA)	0
Formicidae	2.70 (3.23)	1.55 (1.04)	1.00 (0.00)	0
Halictidae	3.57 (3.39)	1.67 (0.90)	2.06 (1.09)	1.33 (0.58)
Ichneumonidae	2.52 (2.01)	1.14 (0.38)	1.78 (1.20)	1.00 (NA)
Megachilidae	2.59 (1.77)	6.11 (4.70)	1.87 (1.55)	1.00 (0.00)
Megaspilidae	1.00 (0.00)	0	0	0
Melittidae	1.20 (0.45)	0	0	0
Mymaridae	1.50 (0.90)	1.78 (1.30)	1.22 (0.44)	1.00 (0.00)
Pteromalidae	1.38 (0.65)	1.67 (1.15)	1.62 (0.74)	0
Scelionidae	1.44 (1.33)	1.25 (0.50)	1.78 (0.83)	2.00 (NA)
Scoliidae	1.43 (1.34)	2.75 (0.96)	1.00 (0.00)	1.00 (NA)
Torymidae	1.00 (NA)	0	0	0
Trichogrammatidae	1.00 (0.00)	1.00 (0.00)	1.20 (0.45)	0
Vespidae	1.41 (0.71)	1.83 (0.41)	2.00 (1.47)	2.75 (2.06)
Mean number	2.64 (3.08)	2.35 (2.61)	1.95 (1.58)	1.56 (1)
% of individuals	61.9	16.4	19.4	2.2

NA indicates that the standard deviation (sd) could not be calculated because only one individual was recorded for that family at that crop development stage.

**Table 4:** Indicator families (IndVal) for trap types.

Families	Group	IndVal	*p* Value
Andrenidae	Blue	0.424	0.005
Scoliidae	White + Yellow	0.482	0.039
Halictidae	Blue + White + Yellow	0.795	0.001
Megachilidae	Blue + White + Yellow	0.691	0.001
Vespidae	Blue + White + Yellow	0.667	0.001
Eulophidae	Blue + White + Yellow	0.664	0.003
Ichneumonidae	Malaise + White + Yellow	0.648	0.007

## Data Availability

The datasets generated during and/or analyzed during the current study are available from the corresponding author upon reasonable request.
